# Genome Sequencing of Polydrug-, Multidrug-, and Extensively Drug-Resistant Mycobacterium tuberculosis Strains from South India

**DOI:** 10.1128/MRA.01388-18

**Published:** 2019-03-21

**Authors:** Sivakumar Shanmugam, Narender Kumar, Dina Nair, Mohan Natrajan, Srikanth Prasad Tripathy, Sharon J. Peacock, Soumya Swaminathan, Uma Devi Ranganathan

**Affiliations:** a ICMR–National Institute for Research in Tuberculosis, Chennai, India; b Department of Clinical Medicine, University of Cambridge, Cambridge, United Kingdom; c World Health Organization, Geneva, Switzerland; d London School of Hygiene and Tropical Medicine, London, United Kingdom; University of Southern California

## Abstract

The genomes of 16 clinical Mycobacterium tuberculosis isolates were subjected to whole-genome sequencing to identify mutations related to resistance to one or more anti-*Mycobacterium* drugs. The sequence data will help in understanding the genomic characteristics of M. tuberculosis isolates and their resistance mutations prevalent in South India.

## ANNOUNCEMENT

Tuberculosis (TB) is the leading cause of death due to a single infectious agent worldwide. As per a World Health Organization (WHO) report, an estimated 10 million people developed TB in 2017 (1). The emergence of strains of multidrug-resistant tuberculosis (MDR-TB) that are resistant to the most effective drugs (rifampin and isoniazid) poses a threat to TB control programs. These constitute 82% of the 558,000 rifampin-resistant cases reported in 2017 worldwide. Among the MDR-TB cases, 8.5% are identified as extensively drug-resistant TB (XDR-TB) and have additional resistance to one of the fluoroquinolones and aminoglycosides. India alone contributed to 2,650 notified cases of XDR-TB in 2017 (1, 2). Early and accurate diagnosis of MDR and XDR-TB followed by appropriate treatment is of critical importance if the number of new cases is to be reduced worldwide. Here, we describe the use of whole-genome sequencing (WGS) technology to define the genetic mutations in isolates from a part of the world that lacks such data and which could contribute to the genetic prediction of a tailored treatment regimen for TB patients in the future.

We report the whole-genome sequence of 16 Mycobacterium tuberculosis isolates from patients in Chennai, India. These have a range of drug resistance patterns that categorize them as MDR, polydrug-resistant (PDR), or XDR. In order to obtain the mycobacterial DNA for WGS, M. tuberculosis strains were isolated from sputum samples of pulmonary tuberculosis patients treated at NIRT, Chennai and grown on Lowenstein-Jensen (LJ) medium, and colonies were scraped from the 3+ growth on the medium into Tris-ethylenediaminetetraacetic acid (TE) buffer (3–5) followed by genomic DNA extraction with a cetyltrimethylammonium bromide-sodium chloride (CTAB–NaCl) extraction method (6). Drug susceptibility of the M. tuberculosis isolates was tested with an automated MGIT 960 instrument as per the manufacturer’s protocol (7, 8). DNA purity and concentration were determined with Nanodrop (9) and Qubit 3.0 fluorometers, respectively (10). Library preparation was performed with the NEXTFLEX DNA library kit as defined by the manufacturer’s protocol (11). Briefly, genomic DNA was sheared to generate fragments of approximately 200 to 600 bp. The fragment size distribution was checked using an Agilent Bioanalyzer (Agilent Technologies) according to the manufacturer’s instructions (12). These adapter ligated fragments were subjected to 10 rounds of PCR with primers provided in the NEXTFLEX DNA sequencing kit. The libraries were quantified using quantitative PCR (qPCR, KAPA library quantification kit, Kappabiosystems) normalized to 1 nM and denatured with 0.1 N NaOH before sequencing with MiSeq v2 cluster chemistry (Illumina). Raw sequence reads were filtered with Trimmomatic v0.36 (13) (quality >20) and assembled with SPAdes v3.11.0 (14) with default parameters, and the contigs larger than 500 bp were filtered for further analysis. The filtered reads were also aligned to H37Rv (GenBank accession number NC_000962) using bwa v0.7.12 (15). The alignment obtained was processed for correction of alignments at indels by the local realignment with a combination of Picard v2.2.4 (http://broadinstitute.github.io/picard/) and GATK v3.5 (16). The variants were identified with SAMtools v1.3.1 (17) and were filtered based on the following parameters: base quality greater than 50, depth greater than 20, mapping quality greater than 30, and alternate allele proportion greater than 75%. The variants were further annotated using a custom Python script (https://github.com/kumarnaren/mtb_vcf_annotator).

Table 1 lists the characteristics of the 16 genomes that were sequenced. The complete length of the assembled genomes ranged from 4,220,229 to 438,963 bp, with coverage depth ranging from 52× to 198×. Examination of the genetic variants revealed the presence of the well-known mutations S315T in *katG* and S450L in *rpoB* (18, 19) to be the predominant mutations in isoniazid- and rifampin-resistant isolates, respectively. Isolates were assigned to lineages 1 to 4 based on the presence or absence of phylogenetic single nucleotide polymorphisms (SNPs) reported by Coll et al. (18). Lineage 1 was dominant in our collection of 16 isolates, which is consistent with our previous reports (3, 5) based on spoligotyping and WGS of drug-susceptible clinical isolates of M. tuberculosis.

**TABLE 1:** Summary of sequence details, drug resistance category, and lineage prediction for M. tuberculosis isolates

NCBI identification no.^*a*^	Assembly accession no.	Coverage depth (×)	Read length (bp)	GC content (%)	*N*_50_ value (bp)	No. of contigs >500	Genome size (bp)	Reference coverage (H37Rv) (%)	No. of tRNAs	No. of rRNAs	No. of coding DNA sequences	Lineage
NIRTX002_PDR	SDOU00000000	74	151	65.58	90,381	103	4,220,229	99.3	44	0	3,997	1
NIRTX003_XDR	SDOT00000000	131	151	65.58	98,656	107	4,375,554	99.2	45	3	4,139	3
NIRTX004_XDR	SDOS00000000	79	100	65.35	57,187	136	4,233,503	96.3	45	2	4,112	4
NIRTX005_XDR	SDOR00000000	80	150	65.37	60,784	140	4,262,450	96.8	45	4	4,163	4
NIRTX006_XDR	SDOQ00000000	75	76	65.59	98,740	114	4,350,302	98.6	45	3	4,119	2
NIRTX007_PDR	SDOP00000000	85	76	65.58	85,411	133	4,389,631	99.5	45	3	4,145	1
NIRTX008_XDR	SDOO00000000	52	76	65.67	114,871	89	4,300,325	97.5	45	1	4,068	4
NIRTX009_XDR	SDON00000000	91	76	65.59	108,252	91	4,379,992	99.3	45	3	4,103	1
NIRTX010_PDR	SDOM00000000	69	76	65.61	72,683	127	4,329,145	98.1	45	3	4,118	2
NIRTX011_XDR	SDOL00000000	60	76	65.59	115,334	96	4,384,136	99.4	45	3	4,113	1
NIRTX015_MDR	SDOK00000000	198	76	65.59	105,690	109	4,366,330	99.0	45	3	4,113	1
NIRTX016_MDR	SDOJ00000000	71	76	65.59	104,750	99	4,378,706	99.3	45	3	4,123	1
NIRTX019_MDR	SDOI00000000	53	76	65.56	97,386	98	4,370,427	99.1	45	3	4,113	1
NIRTX022_MDR	SDOH00000000	174	151	65.61	94,182	101	4,362,069	98.9	45	3	4,136	4
NIRTX024_MDR	SDOG00000000	68	151	65.56	105,728	92	4,383,766	99.4	45	3	4,152	1
NIRTX025_MDR	SDOF00000000	92	151	65.54	25,670	323	4,380,819	99.9	49	8	4,295	1

a MDR, multidrug-resistant TB; XDR, extensively drug-resistant TB; PDR, polydrug-resistant TB.

The sequenced genomes of these isolates will contribute toward an understanding of the genomic characteristics of strains of M. tuberculosis that are prevalent in India, including the genetic mutations that cause resistance. In particular, these data add to knowledge about isolates from South India and provide a resource for future studies of M. tuberculosis in the region.

### Data availability.

The whole-genome sequence reads of the Mycobacterium tuberculosis isolates reported here are deposited in the Sequence Read Archive (SRA) under accession number PRJNA492975. This whole-genome shotgun project (assembly) has been deposited in GenBank under the accession numbers SDOF00000000 to SDOU00000000. The versions described in this paper are accession numbers SDOF01000000 to SDOU01000000.
